# Intestinal Microbiota as a Host Defense Mechanism to Infectious Threats

**DOI:** 10.3389/fmicb.2018.03328

**Published:** 2019-01-23

**Authors:** Simona Iacob, Diana Gabriela Iacob, Luminita Monica Luminos

**Affiliations:** ^1^Department of Infectious Diseases, Carol Davila University of Medicine and Pharmacy, Bucharest, Romania; ^2^National Institute of Infectious Diseases “Prof. Dr. Matei Bals”, Bucharest, Romania

**Keywords:** intestinal microbiota, infection, commensal flora, bacterial enteropathogens, colonization resistance, mucosal immunity, short chain fatty acids

## Abstract

The intestinal microbiota is a complex microbial community, with diverse and stable populations hosted by the gastrointestinal tract since birth. This ecosystem holds multiple anti-infectious, anti-inflammatory, and immune modulating roles decisive for intestinal homeostasis. Among these, colonization resistance refers to the dynamic antagonistic interactions between commensals and pathogenic flora. Hence, gut bacteria compete for the same intestinal niches and substrates, while also releasing antimicrobial substances such as bacteriocines and changing the environmental conditions. Short chain fatty acids (SCFAs) generated in anaerobic conditions prompt epigenetic regulatory mechanisms that favor a tolerogenic immune response. In addition, the commensal flora is involved in the synthesis of bactericidal products, namely secondary biliary acids or antimicrobial peptides (AMPs) such as cathellicidin-LL37, an immunomodulatory, antimicrobial, and wound healing peptide. Gut microbiota is protected through symbiotic relations with the hosting organism and by quorum sensing, a specific cell-to-cell communication system. Any alterations of these relationships favor the uncontrollable multiplication of the resident pathobionts or external entero-pathogens, prompting systemic translocations, inflammatory reactions, or exacerbations of bacterial virulence mechanisms (T6SS, T3SS) and ultimately lead to gastrointestinal or systemic infections. The article describes the metabolic and immunological mechanisms through which the intestinal microbiota is both an ally of the organism against enteric pathogens and an enemy that favors the development of infections.

## Introduction

The human body hosts trillions of microorganisms at the level of the skin and mucous membranes. Together they build a complex microbial community, with multiple metabolic and immune functions that are essential for the survival of the human organism (Lin and Zhang, 2017).The most important representation of this community can be found in the gut as the intestinal microbiota. The gut microbiota is a stable and diverse ecosystem established upon birth and that gradually develops complex digestive and metabolic functions. Along with the intestinal epithelium, the microbiota forms a protective barrier against infectious threats and plays an active role in promoting and maintaining the immune homeostasis during intestinal infections. In order to accomplish these functions, commensal bacteria that colonize the intestinal tract develop symbiotic relationships with the human organism while also competing against microbial pathogens.

The article is centered on the how antiinfectious defense mechanisms intervene during host-microbiota interactions as well as during the direct competition between commensals and pathogens. The review details complex antiinfectious mechanisms ensuring the success of the microbiota in this competition, including quorum sensing (QS), colonization resistance, direct bactericidal activity, cooperation with the immune system and counteracting strategies developed by enteric pathogens to ensure their survival.

## Microbiota and Infectious Threats

The cells of the gut microbiota outnumber the somatic cells by 10–100 times, yet most cannot be cultivated *in vitro*. Molecular techniques have revealed that the flora of healthy individuals contains over 99.1% bacterial species and is mainly represented by 4 phyla, *Firmicutes*, *Bacteroidetes, Actinobacteria*, and *Proteobacteria*, the remaining ones being viruses or members of the *Archaea or Eukarya* domain. At birth the microbiota colonizes the intestinal tract and gradually diversifies so that after 3 years the dominant pattern belongs to anaerobic species *Bacteroidetes, Firmicutes* and especially *Clostridium cluster IV/XIV* (Rajilic-Stojanovic et al., 2007). The small intestine predominantly hosts *Streptococcus, Veillonella* and *Lactobacillus* species, while the large intestine and distal gut contain anaerobic species, mainly *Bacteroides*, *Clostridium*, *Fusobacterium*, and *Bifidobacterium* and facultative anaerobic species like *Escherichia*, *Enterobacter, Enterococcus, Klebsiella, Lactobacillus*, and *Proteus* (Donaldson et al., 2016). The relationship between the gut microbiota and the organism can be symbiotic, mutual or commensal, thus allowing the colonization with protective species (“symbionts”), among which the most beneficial are *Lactobacillus* and *Bifidobacterium* (Walter, 2008; O’Callaghan and van Sinderen, 2016). In certain conditions though, pathogenic or potentially pathogenic microorganisms (“pathobionts”) may trigger inflammatory, oncogenic or infectious diseases. The pathogens that enter the digestive tract induce diarrheal diseases complicated by dehydration and organ failure. In addition, intestinal pathogens release toxins, penetrate through the mucus layer, adhere to the intestinal epithelium, manipulate the intestinal immunity, and sometimes translocate into the systemic circulation (Stecher and Hardt, 2008). The most threatening enteropathogens include: *Shigella; Campylobacter; Yersinia*; and non-typhoid *Salmonella* species associated with inflammatory diarrhea; toxin producing Vibrio *cholerae* and *Escherichia coli* species responsible for secretory diarrhea; and *S. typhi/parathiphy* endotoxin producers causing systemic life-threatening enteric fevers. Among conditional pathogens, two representatives stand out: *Enterococcus species* responsible for endocarditis and *Clostridium difficile*, a frequent cause of post-antibiotic enterocolitis (Perez-Lopez et al., 2016).

The organism opposes enteric pathogens through an “intestinal barrier” composed of 3 interdependent components, namely the intestinal microbiota, the continuous intestinal epithelium protected by the mucus layer and the mucosal immunity. Within this structure, the microbiota restricts the access of pathogens to the intestinal mucosa through specific competitive mechanisms also termed “colonization resistance” along with QS, a network of communication molecules.

## Bacterial Quorum Sensing in the Antimicrobial Defense

Despite the physiological variations and challenging survival conditions in the gut, the microbiota has proved to be an extremely stable structure throughout time. This stability is ensured by QS, an intercellular communication mechanism based on signaling molecules accumulated during bacterial replication and regulated by feed-back (Thompson et al., 2016). These chemical signals referred to as “autoinducers” activate specific receptors and induce bacterial phenotypic changes correlated with adherence, motility and intestinal density of bacteria or with the secretion of protective short chain fatty acids (SCFAs) and antimicrobial peptides (AMPs). QS is employed both by commensals, to ensure gut homeostasis, and by pathogens, to minimize host immune responses and help express pathogenicity. One of these signals is the autoinducer-2 (AI-2), released and recognized by a wide range of commensal bacteria (Pereira et al., 2013). Hence, AI-2 produced by the commensal *R.obeum*, through accumulation and negative feedback, restricts the colonization with the pathogenic *V. cholera* (Hsiao et al., 2014). Enteropathogens (*E. coli, Clostridia, Listeria, and Pseudomonas species*) exploit QS systems against commensals by activating virulence factors, toxin synthesis, sporulation, biofilm formation or by inducing antimicrobial resistance (Chen et al., 2014; Asfour, 2018; Pinheiro et al., 2018). Better knowledge of cell-cell QS signals and the ability to enzymatically block some of the signals (Quorum Quenching) already observed in many plants and demonstrated on animal models could provide alternative solutions in the future for antimicrobial treatment against multiresistant strains.

## Colonization Resistance Against Pathogens

The concept of colonization resistance refers to the ability of commensals to prevent the colonization and overgrowth of intestinal pathogens (Lawley and Walker, 2013). Colonization resistance is a mechanism of intestinal protection following the direct competition between commensals and pathogens for the intestinal niche and nutritive resources.

### Niche Competition: Mucus-Layer Protection

The intestinal epithelium is represented by a monolayer of cells joined through tight junctions which plays a complex role in mucus production and immune response (Rakoff-Nahoum et al., 2004; Kong et al., 2018). Ever since birth, the commensal flora attaches to the intestinal mucus, occupying all available spaces and impeding enteropathogenic colonization (Huang et al., 2011). The mucin-rich mucus layer exhibits O-glycan structures or peptidic motifs that function as receptors for bacterial adhesion, allowing the colonization with health-promoting bacteria such as *Lactobacillus* and *Bifidobacterium* (Kinoshita et al., 2007) and interfering in the metabolism of certain immunoprotective species ( *Bacteroides thetaiotaomicron, Bacteroides fragilis)* (Coyne et al., 2005; Martens et al., 2009; Bergstrom and Xia, 2013). Mucin promotes bacterial clearance, modulates virulence factors and biofilm formation, while also ensuring the survival of commensals in the competition with entheropathogens (Sicard et al., 2017). Mucus thus forms the natural habitat of intestinal symbionts and is among the first defense mechanisms of the intestinal epithelia against bacterial invasion. Mucin competition is used for survival by commensal strains of *E. coli* or *Bifidobacterium* versus pathogenic *E. coli or C. difficile* (Collado et al., 2005; O’Callaghan and van Sinderen, 2016). The probiotics have also been shown to stimulate mucus secretion and intestinal colonization (Collado et al., 2005; Caballero-Franco et al., 2007). Some enteropathogens such as *Samonella* and *Helicobacter pylori* have developed strategies to avoid mucus or overcome mucus receptors such as flagellar motility (*Salmonella, Campylobacter, C. difficile, V. cholerae*)., mucinases secretion (*Salmonella typhimurium, E. coli and S. flexneri, V. cholerae*) or lack of adhesins involved in intestinal adherence. Others have developed mechanisms that anchor them better to the mucus through mucin-binding proteins (*Listeria, H. pylori*) or fimbrial adhesins (*S. enterica*) or activate certain virulence mechanisms, namely type VI or III secretion system (T6SS,T3SS) (e.g., *V. cholerae*) (Branka et al., 1997; Tu et al., 2008; Ganesh et al., 2013; Luo et al., 2014; Liu et al., 2015; Sicard et al., 2017). In this context, mucus plays an important role in bacterial pathogenic mechanisms and displays the potential to become a target for future anti-diarrheal drug therapies (Caballero-Franco et al., 2007).

### Nutrient Competition: Mucus as a Nutritive Resource

Mucins and dietary complex carbohydrates are essential intestinal nutritive resources (Kim and Ho, 2010) to which commensal species have adapted through specific metabolic pathways (Ndeh and Gilbert, 2018). Enteropathogens frequently use nutritious sources offered by commensal species; pathogenic germs that are unable to metabolize these sources are frequently eliminated. Hence, the pathogenic *C. difficile* survives by metabolizing the succinate from the symbiont *B. thetaiotaomicron*, whereas *C. difficile* *mutans* unable to metabolize succinate are eliminated (Ferreyra et al., 2014). Furthermore these metabolic pathways also ensure colonization resistance. Thus, the glycan fucosylations predominantly induced by *Bacteroides* provide a source of nutrition to symbionts and favor their colonization. This mechanism is vital for the colonization of *Bacteroides* species as well as for enteropathogens that require fucosis to express virulence (Coyne et al., 2005; Pacheco et al., 2012; Wands et al., 2015). *S. typhimurium, C. difficile, E. coli, Campylobacter jejuni* still use fucose or other metabolic products of commensal species (sialic acid, succinate) to regulate their expansion and pathogenicity (Ng et al., 2013; Curtis et al., 2014; Dwivedi et al., 2016; Sicard et al., 2018).

However, some pathogenic bacteria may use digestive nutrients that commensals cannot metabolize. Thus, ethanolamine, a carbon and nitrogen source for *S. typhimurium*, *Enterohemorrhagic E. coli* (EHEC), *Klebsiella, Pseudomonas, C. difficile* and *Listeria monocytogenes* cannot be used by most commensal species (Garsin, 2010). EHEC species in particular have developed metabolic pathways for distinct sugar resources, some of which are inaccessible to commensal *E. coli* (Fabich et al., 2008). Interestingly, in the presence of multiple distinct strains of commensal *E. coli*, EHEC could fail to colonize the gut according to mice experiments (Maltby et al., 2013). Diversified diets offering a variety of sugars can provide the necessary resources for some pathogenic germs even during their competition with the commensal flora. *Citrobacter rodentium* is such an example as it requires multiple mono or polysaccharides and has a high rate of colonization. However in the case of a diet based solely on monosaccharides, *C. rodentium* cannot survive the competition with several commensal species (Kamada et al., 2012). As a result, the dietary intake of nutrients plays a significant role in the pathogenesis of enteric infections.

The effective colonization of intestinal niches also requires the ability to elude the immune barrier mounted by the mucosal immunity and the specific antimicrobial compounds of the microbiota, such as antimicrobial peptides, secondary bile acids (BA) and SCFA.

## The Role of Mucosal Immunity

**FIGURE 1:**
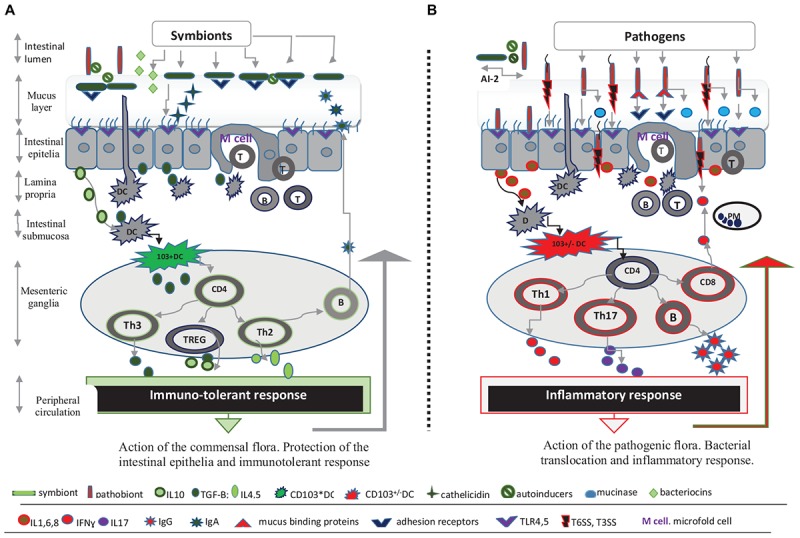
Microbiota and the intestinal barrier against infectious threats. **(A)** Symbionts shape mucosal immunity and protect intestinal epithelia. The commensal flora, intestinal epithelia covered by mucus, and the mucosal immune response form a complex intestinal barrier against infectious threats. The first line of defense is represented by the mucus layer as well as by antimicrobial peptides (cathelicidin), bacteriocins, and intestinal IgA that ensure the integrity of intestinal epithelial cells (IECs). The commensal flora is interconnected through Quorum-sensing signals (e.g., AI-2 inducer) and is anchored to the protective mucus, at a distance from the intestinal epithelia. Thus commensal species reach high densities in mucosal niches and use available nutrient sources contributing to the colonization resistance. The mucosal immunity is best represented by a network of cells and cytokines within the lamina propria, submucosa and mesenteric ganglia that are ultimately modulated by the commensal flora. Commensals activate resident submucosal dendritic cells (DCs) and initiate an immunosuppressive response through NFkB/TGFβ pathway which in turn activates the lymphnode CD103^+^ DCs mesenteric population. These DCs subset have the ability to prime naïve CD4+ T cell lymphocytes from mesenteric ganglia and induce immunosuppressive CD4+ T cells lineages, namely Th3, FoxP3+ (TREG) and Th2-inducing IL10, and TGF-β cytokines. The immune-tolerant response protects IECs and allows symbiotic relationships between the organism and commensal flora. **(B)** Intestinal pathogens activate inflammatory mediators and promote invasion. Pathogenic species possess virulence factors (T6SS or T3SS system, flagella, mucus binding proteins, mucinases, etc.) that enable their mucus diffusion and translocation across IECs. Herein pathogens activate TLR4 and 5 receptors and subsequently induce the release of inflammatory cytokines, IL1, IL6, and IL8. These cytokines further activate the CD103+/-DCs population and either promote CD4 T helper cells polarization into inflammatory Th1, Th17, and CD8-cytotoxic subsets or stimulate B lymphocytes to express IgG, while also reducing the immunosuppressive response. The IgG activity and inflammatory response driven by Th1/Th17-related cytokines, IFN-γ, IL-17, limits the bacterial invasion. The AI-2 inducer produced by certain symbionts can also prevent intestinal colonization with pathogenic species through negative feedback signals. However a significant gut inflammatory response allows bacterial translocation.

The mucosal immune response prevents the invasion of intestinal niches by pathogens and ensures the immune tolerance toward commensal germs (Wershil and Furuta, 2008). In particular, DCs belonging to gut-associated lymphoid tissue are a key population able to regulate mucosal immunity and intestinal homeostasis. Thus, DCs are able to mount a tolerant response by downregulating the NF-kB signaling cascade when DC activation is induced by commensals through either epithelial-cell-derived cytokines (TGF-β, IL10), luminal antigens or microfold cells. Tolerogenic DC from mesenteric lymph nodes also display a DC CD103+ profile that enables the conversion of naïve CD4+T cells toward either of the following immunosuppressive lymphocyte lineages: Th3, FoxP3+(TREG) and Th2 which favors IgA secreting B-cells (Coombes and Powrie, 2008). Additionally, anti-inflammatory cytokines (IL4,5,10,TGF-β) and IgA ensure the protection of the intestinal epithelia and the anchorage of the commensal flora to the protective mucus (Hasegawa et al., 2014). On the other hand, a proinflammatory response ensues when the TLR mediated response generated by pathogens switches the DC tolerogenic profile to the inflammatory CD103-/+ mesenteric DCs. DCs will further upregulate the NF-kB system and stimulate CD4 differentiation into inflammatory Th1, Th17, and CD8-cytotoxic subsets along with IgG secreting B lymphocytes. Furthermore, proinflammatory cytokines (IL6, IL8, IL17, IL21, IL22, and IFN-γ) will recruit neutrophils to the invaded site (Vandamme et al., 2012; Hunter and De Plaen, 2014; Perez-Lopez et al., 2016). The inflammatory response ensures epithelial integrity and prevents the invasion of the intestinal mucosa by *Salmonella, C. jejuni* or other pathogens (Geddes et al., 2011). Dysregulations of the NF-κB pathway could nevertheless induce an excessive inflammatory response and epithelial disruption, promoting translocations of bacteria and toxins (Figure 1).

## Microbiota and Its Direct Weapons, the Antimicrobial Compounds

### Antimicrobial Peptides

Antimicrobial peptides are innate immune effector molecules with bactericidal, anti-inflammatory and anti-endotoxinic properties (Vandamme et al., 2012). Cathelicidin, a vitamin D mediated AMP, favors mucus synthesis and epithelial repair, as well as chemotaxis and T-cell recruitment to the invaded site. The intestinal microbiota facilitates the synthesis of AMP in the intestinal epithelium via a pattern recognition receptor mediated mechanism or indirectly, through its own metabolic products, in particular SCFA (Schauber et al., 2003; Raqib et al., 2012). *B. thetaiotaomicron* and *Listeria innocua* are the main species that induce AMP, especially angiogenins, cathelicidin and several defensines (Hooper et al., 2003; Eckmann, 2005). AMP release can be down-regulated by certain pathogens (*Helycobacter, Schigella*) or up-regulated by probiotics (*Lactobacillus*, *Bifidobacterium)*, explaining the success of the experimental use of probiotics in the prophylaxis of intestinal infections in birds (Sunkara et al., 2012).

### Bacteriocins

The commensal flora produces cell wall-active bactericidal polypeptides generically called bacteriocins. Bacteriocins are produced by *Eubacteria* and some *Archaea* species and are classified according to their peptidic structure (Garcia-Gutierrez et al., 2018). Bacteriocin-producing species are best represented by the families of the *Lactobacillaceae* and *Bifidobacterium* (Hammami et al., 2013). Each bacterial species produces several types of bacteriocins regulated by QS in a cell density-dependent manner (Kleerebezem, 2004). Bacteriocins significantly limit the colonization with multidrug resistant enterococci (Millette et al., 2008), *L. monocytogenes* (Dabour et al., 2009), *C. difficile* (Crowther et al., 2013), *S.aureus* (Park et al., 2007) and other pathogens (Umu et al., 2016). In addition, probiotics also release bacteriocins responsible for the elimination of certain pathogenic species and for the restoration of commensal intestinal communities. Thus, the probiotic strain *E. coli* Nissle-1917 induces anti *Shiga-toxin* bacteriocins (Mohsin et al., 2015) while PA-1 pediocin acts upon *Listeria* species (Dabour et al., 2009). Sactibiotic thuricin-CD, a bacteriocin produced by a strain of *B.thuringiensis*, exhibits inhibitory concentrations against *C. difficile* that are comparable to vancomycin and metronidazole without the former’s toxicity (Mathur et al., 2016). Despite increasing attention, the study of bacteriocins is impeded by unstandardized methods and discordant results on animal models.

### Secondary Bile Acids

Bile acids are essential antimicrobial agents controlled by the intestinal microbiota acting through cell membrane-damage or by activating vitamin D and farnesoid X nuclear receptors involved in the regulation of cathelicidin (Ridlon et al., 2014; Martin et al., 2018).

The liver synthesizes cholic acid and chenodeoxycholic acids, two primary BAs deconjugated by bacteria bile salt hydrolases (BSHs) and converted to secondary BAs, namely deoxycholic acid (DCA) are” to “*Clostridium* and *Bacteroides* are” in the sentence “It has been suggested that…” for meaning. Kindly confirm if this is fine. and lithocholic acid. It has been suggested that BSHs-producing species of *Lactobacillus, Bifidobacterium, Clostridium*, and *Bacteroides* are protected from the toxicity of BA and are advantaged in the competition for survival (Horáčková, 2018). One example is the invasive BSH-producing *L. monocytogenes* strain versus BSH-negative and non-virulent *L. innocua* (Dussurget et al., 2002). Deconjugation is an attribute of Gram positive bacilli, especially of the commensal *Lactobacillus* and *Clostridium* species (e.g., *C. scindens*) and ensures the colonization resistance toward other species (Buffie et al., 2015). *B.bifidum*-induced DCA diminishes the pathogenicity of certain germs by targeting virulence-associated T6SS or T3SS effectors, used by toxigenic enterobacteria species and *V. cholerae* (Urdaneta and Casadesús, 2017). Furthermore, the absence of DCA-producing commensals allows the expansion of pathogens as in the case of *C. difficile* enterocolitis (Kochan et al., 2018). Increasing the level of BAs through therapy or diets favors the expansion of species involved in BA deconjugation (*Firmicutes* and *Clostridium* in particular) along with the inhibition of *Bacteroidetes* and *Actinobacteria.* Nevertheless, certain invasive species such as *Salmonella* can survive for prolonged periods despite high concentrations of BAs (Urdaneta and Casadesús, 2017). Considering that excessive BAs, especially DCA, are involved in the development of cholesterol gallstones and colon cancer, the intestinal flora can play an additive role to these complications. Concurrently, low concentrations of BAs are correlated with reduced antimicrobial function, favoring Gram negative bacterial overgrowth and translocation (Ridlon et al., 2014).

### Short Chain Fatty Acids

Short chain fatty acids are saturated aliphatic organic acids consisting of a chain of 2–6 carbon atoms and a carboxylic acid moiety. SCFAs result from fermentation processes initiated by the gut and perform immune, anti-infectious and metabolic roles. There are three main types of SCFA: acetate, propionate and butyrate. Butyrate is mostly produced by *Firmicutes* in the colon, while acetate and propionate produced by *Bacteroidetes* strains predominate in the systemic circulation (Cummings et al., 1987).

**FIGURE 2:**
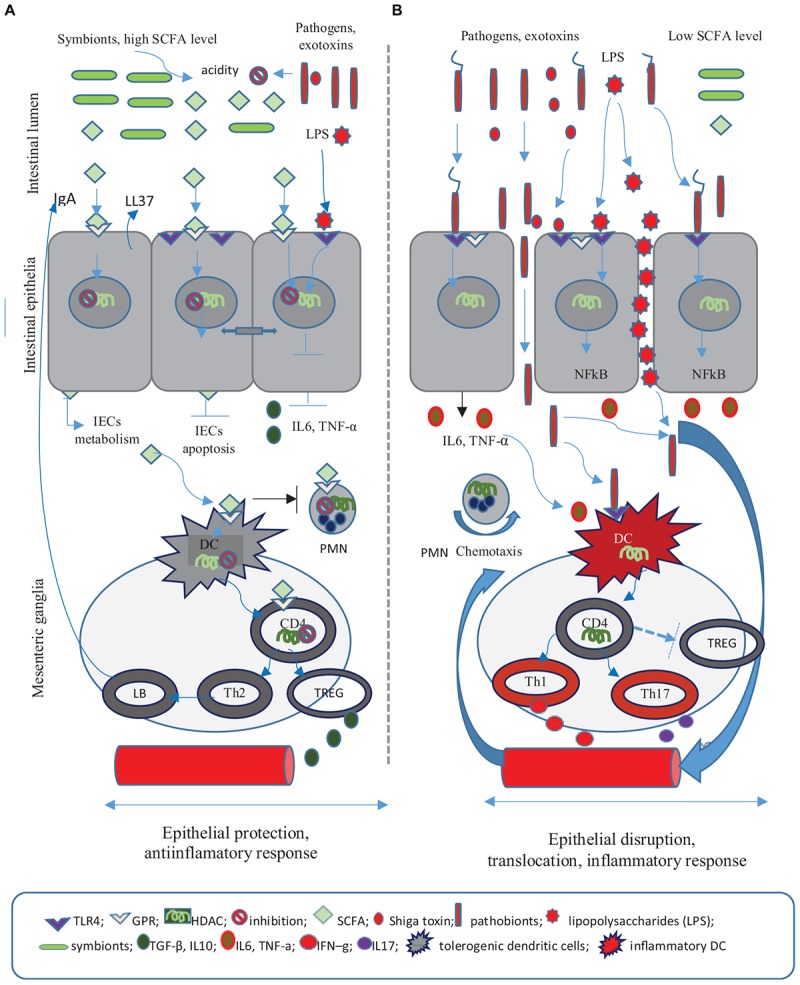
The role of short chain fatty acids (SCFA) in the anti-infectious defense. **(A)** SCFA are fermentation products of the commensal flora and exert anti-inflammatory and trophic roles on the intestinal epithelial cells (IECs). SCFA elicit effects on IECs metabolism increasing colonic acidity and preventing the colonization with pathogenic germs. SCFA play a significant role in the preservation of tight epithelial junctions through the activation of HIF factor and by decreasing enterocyte apopotosis. As a result SCFA decrease epithelial permeability. At a cellular level SCFA activate specific G protein-coupled receptors (GPR), modulate the chromatin through histone deacetylase (HADC) inhibition, supress the nuclear factor (*NF*)-κB and reduce the release of pro-inflammatory cytokines (IL6, TNF-α). SCFA regulate the inflammatory response by promoting tolerogenic dendritic cells (DCs), IL-10-producing T-helper cells (TREG), and Th2 response including *B-*cell IgA production. The immune role of SCFA relies on their ability to induce an anti-inflamatory response and to stimulate antimicrobial molecules, namely cathelicidin (LL37) and intestinal IgA. Consequently SCFA offer protection to IEC and decrease the risk of bacterial invasion and toxin translocation. **(B)** The absence of SCFA leads to epithelial breaches and translocations of bacteria and lipopolysaccharides (LPS). The inflammatory response involves the following: the activation of toll-like receptor 4 (TLR4) by pathogenic flora, HDAC activity, the (NF)*-*B transcription, DCs stimulation which promotes mesenteric Th1/Th17 cell differentiation, the release of pro-inflammatory cytokines IL6, TNF-α, IFN–γ, IL17, and neutrophil migration.

SCFAs are GPR41, GPR43, and GPR109 membrane receptor ligands with immunomodulatory functions. These receptors are located specifically in the intestinal tract but can also be found in leukocytes, hematopoietic tissues (GPR43) or in the enteric nervous system, portal circulation and gut–brain neural circuit (GPR41). GPR109 mainly expressed in the colon epithelia responds to butyrate only. At a cellular level, SCFAs protect the intestinal epithelium and reduce bacterial translocation by stabilizing the hypoxia-inducible transcription factor (Kelly et al., 2015). SCFAs also act as epigenetic regulators of the local and systemic inflammatory response. Thus SCFAs serve as histone acetyltransferases activators or histone deacetylase inhibitors (HDACi) that modulate transcriptional factors (e,g NF-κB, MyoD) (Liu et al., 2012; Koh et al., 2016; Chen et al., 2018). Butyrate, the most potent HDACi, has been shown to modulate the genetic expression of over 19.000 genes in a human colonic epithelial cell line (Daly and Shirazi-Beechey, 2006). SCFAs acting as HDAC is also exert divergent immunologic responses depending on the cell type and its degree of activity and HDACi type (Villagra et al., 2010). SCFAs can thus regulate Th1/Th2 cytokines, often favoring an anti-inflammatory response (DC with a tolerogenic profile, the production of Foxp3^+^(TREG) cells, IL10 release, NF-kB suppression). In addition, SCFAs inhibit inflammatory responses mounted by polymorphonuclear cells, lymphocytes or tissue phagocytes (Figure 2). However, once an inflammatory process has already been initiated, SCFAs further intensify this process.

The action of SCFAs on T lymphocytes depends on their activation – activated T lymphocytes favor SCFA suppression of FoxP3+TREG cells, while decreased T cell activation induces FoxP3+(TREG) cell expansion. Furthermore, in the presence of an infection and of subsequent T cell lymphocyte activation, SCFAs promote the differentiation of Th1 and Th17 and stimulate both effector (pro-inflammatory) and FoxP3+(TREG) cell lines. Still, in the absence of an acute infection SCFAs induce cell production of IL10 and immune tolerance (Cavaglieri et al., 2003; Furusawa et al., 2013; Figure 2).

Short chain fatty acids play a trophic role on the intestinal mucosa, enable the proliferation of normal colon cells and maintain the integrity of epithelial tight junctions. By decreasing epithelial permeability, SCFAs oppose bacterial and LPS translocations. Thus the acetate produced by *Bifidobacteria* inhibits the apoptosis of epithelial cells and prevents gut-blood EHEC*-Shiga* toxins translocation (Fukuda et al., 2011). SCFAs interfere with the lipid and glucose metabolism in the liver and various tissues and represent an energy source for gut epithelia. In addition, SCFAs acidify the intestinal environment further preventing the colonization with pathogenic bacteria (den Besten et al., 2013). Thus *Clostridium* butyrate producers (especially *Lachnospiraceae, Ruminococcaceae*, and *F.prausnitzii*) impede the colonization with *Salmonella*, *C. difficile*, and *Campylobacter species* (Goepfert and Hicks, 1969; Van Deun et al., 2008; Antharam et al., 2013; Rivera-Chávez et al., 2016). As a result, pathogenic species will colonize locations where the pH is convenient for the expression of virulence factors, such as the colon for *Shigella* or the ileum and jejunum for the invasive EHEC and *S. tiphy/S. typhimurium* (Speelman et al., 1984; Tazume et al., 1990). On the other hand SCFAs and particularly butyrate, modulate the expression of cellular receptors (e.g., EHEC*-Shiga* toxin receptors), virulence factors (T3SS) or epithelial adherence factors of some enteropathogens (Nakanishi et al., 2009; Tobe et al., 2011; De Nisco et al., 2018). Consequently, some commensal species may support pathogenic bacteria via SCFA release, while others strengthen the barrier function of the intestinal epithelium and prevent translocations or colonization with bacterial pathogens. These observations highlight the idea that the simple presence of SCFAs is not sufficient to balance the gut-microbiota relationship and that further data is needed on SCFA producing probiotics and their administration.

## Conclusion

The intestinal microbiota is a complex and stable microbial community whose symbiotic relationship with the human organism is crucial for gut homeostasis and colonization resistance against intestinal pathogens. The effective colonization by pathogens is precluded through the competition with commensal flora for the same intestinal niche and nutritional resources, as well as by specific antimicrobial products and mucosal immune response. Pathogens employ multiple strategies to adapt to this hostile environment such as the expression of various virulence factors, structural changes or the manipulation of the QS system. Any changes involving the structure, stability or functional activities of intestinal microbiota could have long-lasting effects and favor intestinal and systemic infections.

## Author Contributions

SI, DI, and LL contributed equally to the acquisition, analysis, and critical revision of the article. All authors had given their consent for the publication and agreed to be responsible for the accuracy and integrity of the article.

## Conflict of Interest Statement

The authors declare that the research was conducted in the absence of any commercial or financial relationships that could be construed as a potential conflict of interest.
